# An Interactive Text Message Survey as a Novel Assessment for Bedtime Routines in Public Health Research: Observational Study

**DOI:** 10.2196/15524

**Published:** 2020-12-21

**Authors:** George Kitsaras, Michaela Goodwin, Julia Allan, Michael Kelly, Iain Pretty

**Affiliations:** 1 University of Manchester Manchester United Kingdom; 2 University of Aberdeen Aberdeen United Kingdom; 3 University of Cambridge Cambridge United Kingdom

**Keywords:** digital technologies, mobile health, child, well-being, development, assessment, bedtime routines, P4 health care, text survey

## Abstract

**Background:**

Traditional research approaches, especially questionnaires and paper-based assessments, limit in-depth understanding of the fluid dynamic processes associated with child well-being and development. This includes bedtime routine activities such as toothbrushing and reading a book before bed. The increase in innovative digital technologies alongside greater use and familiarity among the public creates unique opportunities to use these technical developments in research.

**Objective:**

This study aimed to (1) examine the best way of assessing bedtime routines in families and develop an automated, interactive, text message survey assessment delivered directly to participants’ mobile phones and (2) test the assessment within a predominately deprived sociodemographic sample to explore retention, uptake, feedback, and effectiveness.

**Methods:**

A public and patient involvement project showed clear preference for interactive text surveys regarding bedtime routines. The developed interactive text survey included questions on bedtime routine activities and was delivered for seven consecutive nights to participating parents’ mobile phones. A total of 200 parents participated. Apart from the completion of the text survey, feedback was provided by participants, and data on response, completion, and retention rates were captured.

**Results:**

There was a high retention rate (185/200, 92.5%), and the response rate was high (160/185, 86.5%). In total, 114 participants provided anonymized feedback. Only a small percentage (5/114, 4.4%) of participants reported problems associated with completing the assessment. The majority (99/114, 86.8%) of participants enjoyed their participation in the study, with an average satisfaction score of 4.6 out of 5.

**Conclusions:**

This study demonstrated the potential of deploying SMS text message–based surveys to capture and quantify real-time information on recurrent dynamic processes in public health research. Changes and adaptations based on recommendations are crucial next steps in further exploring the diagnostic and potential intervention properties of text survey and text messaging approaches.

## Introduction

### Background

Capturing high quality and quantity of data is the cornerstone of every successful research project. Most research studies, especially those focusing on behavioral, psychosocial, and wider public health research, use traditional approaches including paper-based questionnaires, interviews, and surveys [1]. These techniques can create barriers to participation for specific population groups, while increasing risk to the quality of data (ie, higher rates of drop out and lower response rates) [1-3]. Easier access, higher uptake, and better use of new technologies, especially mobile phones, create the potential of shifting a number of research-related activities away from traditional approaches [1-3]. In recognition of this shift, organizations around the globe, including the US Food and Drug Administration, recommend electronic capture of data for clinical trials, instead of traditional paper-based methods [4]. Moreover, research conducted by pharmaceutical companies, among others, involves considerable use of mobile-based technologies (37%) in clinical trials [1]. Finally, current trends in health care in the era of the rising P4 (predictive, preventive, personalized, and participatory) model demonstrate the need for innovative, cost-effective, and user-oriented approaches [5].

Currently, the use of mobile phones for communication and entertainment is very high, with approximately 93% of the population owning a mobile phone in the United Kingdom [5]. The use of text messages is also high, with an average of 100 text messages sent per mobile phone subscription per month in the United Kingdom [5]. Unlike many other new technologies, people from low socioeconomic backgrounds and varying ethnicities have similar access to mobile technology as the rest of the population [6]. Low income and minority groups not only show similar rates of using mobile phones but also report a higher percentage of text messaging than other groups [5,6]. Additionally, mobile phone use is the highest among less educated adults and those who rent or frequently change their address [7]. Therefore, in research, mobile phone–based text surveys may represent an important tool for accessing, with minimum effort and intrusiveness, a large number of participants, while obtaining high quality and quantity of data from individuals, including those in traditionally “hard to reach” groups.

Assessment of bedtime routines in families with young children represents an area where text surveys may be implemented in order to gain a better understanding of the fluid and dynamic processes involved. Bedtime routines have shown important associations with a variety of aspects of child development and well-being, especially quality of sleep, dental health, parental psychoemotional well-being, attitude toward learning, and cognitive development [8-15]. Despite evidence highlighting their importance, there is a clear lack of a reliable, flexible, innovative, and user-friendly method of assessment for bedtime routines [16,17].

The main approach when assessing bedtime routines has involved paper-based questionnaires and diaries [18]. Only few studies involved real-life capture of bedtime routines via video recording, possibly owing to the intrusiveness of this method and the associated ethical and legal implications [18]. When using paper-based questionnaires with a retrospective design, the possibility of recall and desirability bias is always present. With routines being incredibly variable and dynamic and with special consideration for intrusiveness and the likelihood of bias, it is important to approach the entire notion of bedtime routine assessment from a different angle. Broader research into families and children has utilized different methodological approaches, such as the ecological momentary assessment, that go some distance in addressing recall bias [19]. However, for most of these approaches, there is a lack of an interactive feature, limiting their dynamic scope.

The proposed novel perspective should use innovative user-friendly technologies that will allow for greater quantity and better quality of data on bedtime routines while minimizing intrusiveness and disruption to participants. Bedtime routines present an area of particular interest for this approach given their dynamic nature that necessitates real-time information collection. If this methodological approach functions in the context of this particular dynamic and repetitive behavior, the same approach can be used in a variety of other research areas.

### Objectives

This study aimed to (1) examine the best way of assessing bedtime routines in families and develop an automated, interactive, text message survey assessment delivered directly to participants’ mobile phones and (2) test the assessment within a predominately deprived sociodemographic sample to explore retention, uptake, feedback, and effectiveness.

## Methods

### Overall Process

**Figure 1:**
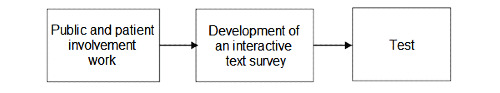
Stepped approach for developing the intervention.

The study followed a series of steps, as presented in Figure 1. All steps were necessary since they informed the next stage of the study with important findings mapping back to the two main objectives.

### Public and Patient Involvement

Public and patient involvement (PPI) work, prior to beginning the overall study, was completed during two separate visits to two preschool centers. The targeted sample for the PPI work was parents with children between the ages of 3 and 5 years. During the visits, a total of 15 mothers (no fathers attended the centers during the PPI work) with their children were approached. All mothers approached agreed to take part in the session, and mothers were approached at random. The mothers were asked questions (on a one-to-one basis) about their views on the best way of assessing their children’s bedtime routines as a recurrent dynamic health-related behavior. As compensation for their time, each family received toothpaste and toothbrushes for both adults and children, and a 2-minute toothbrushing timer. Multimedia Appendix 1 presents an overview of the questions and results from the PPI session.

### Development of an Interactive Text Survey

**Figure 2:**
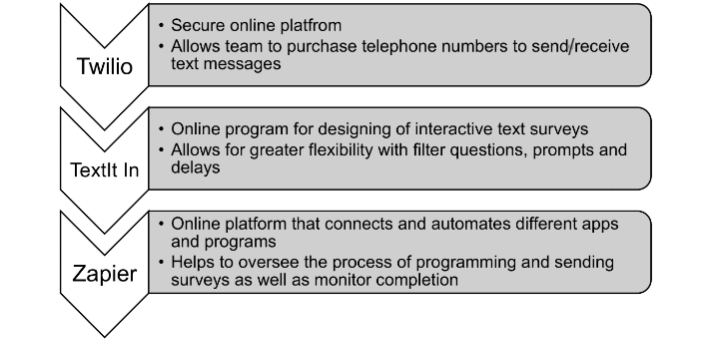
Readily available platforms used in redesigning the text-survey.

Following the completion of the PPI work, an interactive text survey was chosen as the most appropriate method for assessing bedtime routines in families with young children. In total, three readily available software and online platforms were used for the design of the study. Figure 2 presents an overview of how the platforms worked alongside one another to produce the intervention.

Using the idea of an ideal bedtime routine, as described by previous studies [18] and expert opinions, the assessment of bedtime routines focused on the following five target areas: (1) consistency (determined as a child going to bed within a space of an hour every night), (2) tooth brushing, (3) snacks/drinks before bed, (4) use of electronic devices before bed, and (5) book reading. These areas have been consistently included in bedtime routine studies as the most important for child well-being and development [18].

Parents predetermined the exact time they would prefer to receive the text survey; however, there was an option to delay the completion of the survey by 30 minutes or 1 hour, or opt out for that night. Detailed instructions on the appropriate answers for each question were provided using examples of accepted answers, and if someone responded incorrectly, a clarification text was sent to them immediately to help them with their responses. Failure to reply following the instructions in two consecutive attempts led to the end of the survey for that night.

The questions in the interactive text survey were both open-ended and closed. Filter questions (yes/no) led participants to different types of questions. Depending on their replies in filter questions, parents received different responses to strengthen the interactive features of the assessment. Overall, the minimum number of questions to answer each night was eight and the maximum number was 10. All participants were entered into the system via an electronic platform using unique IDs, and all data were managed through secure, university-based, password-protected computers. Table 1 presents a summary of the key characteristics of the interactive text survey as delivered to participants. The overall structure of the interactive text survey is presented in Multimedia Appendix 2.

**Table 1:** Characteristics of the interactive text survey developed following public and patient involvement work.

Characteristic	Interactive text survey value
Minimum number of questions	8
Maximum number of questions	10
Open questions	Yes
Closed questions	Yes
Question on current parental mood	Yes
Question on self-assessed bedtime routine	Yes
Questions on key bedtime routine activities (eg, tooth brushing)	Yes
Opportunity to delay completion	Yes
Opportunity to opt out on a nightly basis	Yes
Predetermined time to receive assessment	Yes
Number of nights receiving assessment	7
Average completion time (min)	2
Activation of a short code for free-of-charge replies	No

The research team opted out from activating a “short code” that would have allowed participants to reply free of charge. The charge that applied to each reply was made clear to participants at the beginning of the study. The charge per text message reply was dependent upon the mobile phone contract and the provider, with most providers in the United Kingdom providing free unlimited text messages as part of their normal packages [5]. For those who do not have a mobile phone monthly package, a simple text message costs between 1 pence (p) and 10 p per text message depending on the provider [5].

### Test Study

#### Recruitment and Eligibility

Recruitment took place between February and July 2018. Participants were approached during their routine appointments in general dental practices. The exclusion criteria were as follows: (1) not having English proficiency, (2) not owning a mobile phone, and (3) having only children under the age of 3 or over the age of 7 years. Participants were informed about the requirements of the study and asked to provide consent. During recruitment, each participant was informed about the compensation that they would receive at the end of the study in the form of online shopping vouchers. The compensation for their time in the study was a maximum of £10. Early withdrawal affected the amount of compensation.

#### Sample

In total, 200 parents were recruited. Parents had a mean age of 34.6 years (SD 5.01), with the youngest being 25 years of age and the oldest being 46 years of age. The vast majority of participants were female, with only 12.0% (24/200) male. Most families had only one child (130/200, 65.0%), with 5.0% (10/200) of families having three or more children. All the children were between the ages of 3 and 7 years. In total, 48.0% (96/200) of the sample had a white ethnic background, 39.5% (79/200) had an Asian/British-Asian ethnic background, and 12.5% (25/200) had a Black/Black British/Caribbean ethnic background. The vast majority (156/200, 78.0%) of participants lived in deprived areas. Deprivation was determined using the index of multiple deprivation (IMD), where higher scores (and quintiles) reflect higher deprivation. For the study, participants had an average IMD score of 41.83 (SD 16.43) and a maximum IMD score of 79.65, which was double the threshold for the fifth IMD quintile.

#### Data Collection

All participants received the interactive text survey on their mobile phones at a predetermined time for a total of seven consecutive nights. As described above, the assessment focused on their bedtime routine activities with a combination of open and closed questions. Feedback was collected using an automated feedback system utilizing the text survey. This system, like the bedtime routine assessment, included both open and closed questions for the experience of each parent. Finally, data relating to uptake, response, and retention rates were collected through an electronic platform (TextIt).

#### Data Analysis

All data were transferred and coded in SPSS (IBM SPSS Statistics for Macintosh, Version 25.0) for analysis. Data analyses focused on (1) bedtime routine activities with data collected from the interactive text survey, (2) feedback data regarding user experience, and (3) uptake, retention, and response rate information.

### Ethical Approval and Consent to Participate

The study in its entirety, including consent forms and all study materials, was previously approved by the Health Research Authority (Integrated Research Application System [IRAS]; ID: 235385; Tyre and Wear South Committee). All participants accepted anonymized use of their data for further analyses and subsequent publication during consent. Written consent was obtained during recruitment. We obtained consent to publish from the participants (or legal parents or guardians for children) to report individual patient data.

### Availability of Data and Materials

The data sets used and/or analyzed in this study are available from the corresponding author on reasonable request.

## Results

### Uptake, Retention, and Response Rates

Of the 200 participants, 185 completed data collection, resulting in an overall 92.5% retention rate over the 7-day period. A total of 11 participants failed to reply to all of the text surveys, and only four participants opted out of the study after providing replies to at least one night of text surveys using the automated opt-out function.

From the 185 people who completed data collection, there was an average response rate across all nights of 87.0% (161/185). There was a steady decrease in the response rate per night during the study, with the first three nights showing response rates over 90%, while the last two nights of the assessment showed response rates below 80%. This may reflect fatigue with the assessment over time. On average, participants replied to at least 5.5 nights of text surveys. The majority (80/185) of participants replied to six nights, while 62 participants replied to all seven nights, 39 replied to five nights, and four replied to only four nights of the text survey. When participants received the text survey and engaged by replying to it, they completed the full survey. Once a participant started the assessment, he/she continued until the last question, resulting in a 100% completion rate per survey, with no missing data associated with noncompletion. Table 2 provides an overview of the completion of the assessment each night.

**Table 2:** Overview of assessment completion each night.

Night	Completion value (N=185), n (%)
1	176 (95.1%)
2	174 (94.1%)
3	169 (91.4%)
4	164 (88.6%)
5	157 (84.9%)
6	146 (78.9%)
7	138 (74.6%)

### Feedback From Users

In total, 114 participants provided anonymized feedback. The majority (99/114, 86.8%) of participants enjoyed their participation in the study, with an average satisfaction score of 4.6 out of 5 and no score below 3 based on the automated feedback system we deployed at the end of the study. The vast majority (107/114, 93.8%) of participants reported high satisfaction scores in receiving and replying to the text messages for seven nights. The average satisfaction score was 4.3 out of 5, and again, no score was below 3. Only a small number (5/114, 4.4%) of participants reported problems caused to their bedtime routines from receiving and replying to the text messages every night for seven nights in total. The low number of people who reported problems may highlight the limited intrusiveness of text messages in assessing behaviors. Participants found the text messages and the questions asked through them to be extremely easy to understand, with an average satisfaction score of 4.9 out of 5. All participants would recommend using text surveys for assessing bedtime routines in future research. The majority (63/114, 55.3%) of those who provided feedback supported the development of a bedtime routine text message support system for those who struggle with their bedtime routines. Finally, a marginal majority (59/114, 51.8%) of participants reported being helped by the nightly text messages in remembering what to do during their bedtime routines.

### Effectiveness in Assessing Dynamic Behaviors

During the study, participants replied to a total of 1125 text surveys, generating 9157 unique data points. On average, each participant generated 50 unique data points by replying to an average of nine questions per night during the course of the study. The deployment of the interactive text survey allowed for a more in-depth observation of bedtime routine activities in families with young children. A small majority (95/185, 51.4%) of participants reported brushing their children’s teeth every night, while only a small percentage (2/185, 1.1%) reported never brushing teeth before bed. With regard to diet, 57.8% (107/185) of participants reported allowing food and/or drinks the hour before bed at some point during the week. A total of 24.9% (46/185) of participants read to their children every night of the week, 9.2% (17/185) never read or shared a book with their children during the course of the study, and 29.2% (54/185) read or shared a book with their children for at least half of the nights. Finally, with regard to the use of electronic devices the hour before bed, 13.5% (25/185) of parents allowed electronic devices to be used the hour before bed.

## Discussion

### Principal Findings

Overall, the interactive text survey assessment of a recurrent dynamic behavior provided a large quantity and good quality of data on the targeted behavior. It managed to keep participants engaged throughout the duration of the study with limited drop out. Finally, the interactive text survey created a user-friendly nonintrusive experience for participants, as reflected in their feedback (Table 3).

**Table 3:** Key results of the study.

Metric	Value, n (%) or score/number
Retention (N=200)	185 (92.5%)
Response (overall) (N=185)	161 (87.0%)
Overall feedback score (scale 0-5)	4.6
Easiness of use score (scale 0-5)	4.9
Reported problems to routines from receiving the assessment (N=114)	5 (4.4%)
Recommended text surveys for future assessments (N=114)	114 (100%)
Supported development of a text messaging bedtime routine intervention (N=114)	63 (55.3%)
Average unique data points	50

The benefits of administering an interactive text survey assessment for recurrent dynamic behaviors can be divided into the following three areas: (1) limiting recall bias, (2) securing higher volumes of data, and (3) providing a better experience for participants. Recall bias can have a potentially detrimental effect on research findings by either underestimating or overestimating the true effect of a given behavior [20]. Recall bias can be affected by a number of areas, with time elapsing between the targeted behavior and assessment of that behavior being one of the most prominent [21]. In traditional assessments, including questionnaires, with a retrospective approach (in some cases, up to a month after the targeted activity), recall bias has the potential to influence both the quality and quantity of collected data owing to the time lapsed [20]. In recurrent and dynamic behaviors, like bedtime routines, with a fluid nature, a narrow timeframe between the activity and assessment of the activity is crucial to be able to capture and investigate their intricate nature. With the development and utilization of approaches that capitalize on narrow timeframes, recall bias can potentially be eliminated.

As for the second area (securing high volumes of data), the deployment of the text survey assessment for bedtime routines led to a considerably higher response rate than with traditional questionnaires [22]. Additionally, once participants started answering the survey on a given night, they completed it without stopping midway or missing any of the questions. This resulted in an average of 50 unique data points. High response rates in conjunction with multiple data points over a short period of time resulted in wider flexibility when conducting further analyses. Finally, with respect to providing a better experience for participants, across all questions on the feedback form, most participants reported no issues and reported a high satisfaction rate. Moreover, when asked at the end of the study, every participant showed a clear preference for a text survey–style assessment against other measures, including questionnaires and video recordings. Across health-related research and intervention studies, there is considerable variation in retention rates, with some studies reporting retention rates as high as 97% and as low as 56% [23-25]. Approaches, such as interactive text surveys, should be further explored and utilized as an alternative method in achieving better user engagement and retention.

In this study, text surveys were used primarily for data collection as an alternative to traditional questionnaire and paper-based assessments for bedtime routines. As discussed, utilization of text surveys and text messages is not limited to data collection, with multiple other functionalities from recruitment to interventions [1,25]. Their increasing availability in conjunction with higher use of mobile phones across all age and demographic groups presents a great opportunity for harnessing their wide spectrum of applications in both research and clinical settings. Multiple organizations like the National Health Service (NHS) in the United Kingdom now deploy text messages and text surveys to contact and remind patients of their appointments and/or to gather feedback about services [26]. These examples showcase in practice how mobile phone–based text surveys and text messages can be used in a reciprocally beneficial relationship where service users or participants and organizations are mutually benefited. Solely for clinical applications, today’s demanding, dynamic, and highly variable health care needs present a great opportunity for mobile phone–based text messages given their cost effectiveness, high adaptability, and flexible format [27-29]. This opportunity is also relevant for pediatric populations given their clear preference for tailored, technology-based, and interactive programs such as mobile phone–based text messages and surveys [29].

Finally, medicine and health care as a whole are slowly but steadily moving to a personalized model of care, as shown by the recent P4 model [30,31]. This model emphasizes the importance of transforming the service from a reactive to a proactive one with regard to disease and care [32]. Even though the model is mainly focused on clinical and long-term conditions, including diabetes, cancer, and cardiovascular diseases, the same approach could possibly be used in a variety of health-related, well-being–related, and development-related behaviors. The P4 model of medicine and health care is driven by the evolution, expansion, and merging of the following three megatrends: (1) systems biology/systems medicine, (2) digital technologies for health care, and (3) consumer-driven health care [30]. Therefore, text message–based applications, including text surveys, can be active components in the era of P4 medicine and health care owing to their versatility, low cost per participant, ease of use, high personalization, and adaptation that transcends cultural, linguistic, and demographic boundaries.

### Limitations

The utilization of a stepped approach in designing, refining, and redeveloping an interactive text survey helps to minimize its limitations. However, there are several areas where limitations are evident. One of the most important is with regard to the risk of bias, especially desirability bias. As with every assessment that relies on self-reported data, desirability bias cannot be fully excluded. Moreover, as highlighted by the comments made by several participants, despite attempting to only assess bedtime routines, the text survey might have acted as an unintended intervention, leading to changes in some family routines. This is not necessarily a negative limitation, since this type of feedback allowed for the consideration of intervening properties regarding text surveys and text messaging for bedtime routines. Finally, another limitation to consider concerns the lack of a reference measure for assessing bedtime routines alongside the utilization of the interactive text survey.

### Recommendations

Despite the success of the approach, necessary changes and recommendations for future use are vital in further exploring the benefits of text surveys in assessing health-related behaviors. These changes can focus on both further improving user interaction with the system and maintaining an overall good user experience. Specific changes include (1) provision for a number to call when and if issues or difficulties arise during the completion of the text survey and (2) provision for free-of-charge replies by activating a “short code,” especially for research focusing on deprived socioeconomic areas and populations. Moreover, based on the feedback of some participants and on this particular area of research, it might be important to consider the development and examination of a text message–based bedtime routine intervention for achieving and sustaining better routines for families with young children.

### Conclusion

The results of this study showed the potential for deploying text surveys within public health research as an attempt to capture real-time information on dynamic and highly variable recurrent behaviors that can have subsequent implications with regard to development and well-being. It also demonstrated that text surveys can be reliable alternatives for capturing data when compared with traditional methods or other technologies, possibly owing to their nonintrusiveness and generally easier user interface. Overall, text surveys and text messages are emerging as valuable alternative routes for capturing data and delivering interventions in wider public health research.

## Appendix

Multimedia Appendix 1 Public and patient involvement questions and results.

Multimedia Appendix 2 Branching flowchart for the text survey.

## Abbreviations

IMD: index of multiple deprivation
P4: predictive, preventive, personalized, and participatory
PPI: public and patient involvement
