# Supplementary material for: An Interactive Text Message Survey as a Novel Assessment for Bedtime Routines in Public Health Research: Observational Study
Source: JMIR Public Health Surveill. 2020 Dec 21;6(4):e15524. doi: 10.2196/15524 (PMC7781795; doi:10.2196/15524)
Supplement: Multimedia Appendix 1 [file publichealth_v6i4e15524_app1.pdf]

Patient and Public Involvement

**Participants**	15
**Gender**	Female = 15
**Age of children**	Youngest 1, oldest 18<br>Average age = 4
**Other characteristics**	Majority Muslim<br>Majority housewives, unemployed or part-time employed<br>Similar SES (=low to middle)

Question	Summary of responses
**Q.1.** ***If you had to chose one option for assessing your bedtime routines, which one will it be and why? Is there any other option-method that comes to mind?***	Text message = 14, important to be without charge<br><br>Online form = 14 as second option<br><br>Paper-based form = 1 because she does not have access to laptop or phone<br><br>Interactive board = may be more complicated and they believed that it might be more difficult to remember and follow on a daily basis
Q.2. If someone asked you to observe your bedtime routines for one or more nights how would you feel about that? How do you think the presence of a stranger affect your family's function?	Against observation = 15, they considered it intrusion of their privacy and they stated that the presence of a stranger in the house will alter normality and affect bedtime routines
Q.3. What is your opinion for the use of video or audio diaries of bedtime routines where you are asked to record your family's practices over a period of time?	Against video/audio diaries = 15, they considered them to be more complicated and in violation of their privacy
Q.4. . How would you feel if you and your child had to complete a set of executive function assessments involving questionnaires and a basic interactive element? Would you be concerned if someone assessed your and your child's executive function?	No problem with EF assessments = 15, but it was important for all of them to have clear knowledge and information beforehand in order to be reassured and less stressed

Q.5. How does this structure (2 visits and week-long bedtime routine assessments) sound?	No issues with proposed structure = 15
Q.6. Based on your family timetables; will it be difficult to have two visits with both parents present? If so, will flexibility from the researcher's end and availability for evening visits make it easier for both parents to be present?	Father will not be willing to participate = 4, because the father has limited participation in child-related activities in general<br><br>Father will not be able to participate = 8, because of father's work schedule and the limited amount they spend at home<br><br>Father will be probably able to participate = 2, if there is enough flexibility from the researcher's end (i.e. evening or weekend visits)<br><br>Father will definitely participate = 1
Q.7. Will the prospect of compensation alleviate concerns and make taking part in the study more attractive? Will £20-25 vouchers be a good motivator to take part and engage fully in the study?	Positive response to compensation = 15<br><br>Belief that compensation may increase the likelihood of paternal participation = 8/12 (3 already stated that the father will likely or definitely participate)
