# Supplementary figures and images for: An Interactive Text Message Survey as a Novel Assessment for Bedtime Routines in Public Health Research: Observational Study
Source: JMIR Public Health Surveill. 2020 Dec 21;6(4):e15524. doi: 10.2196/15524 (PMC7781795; doi:10.2196/15524)

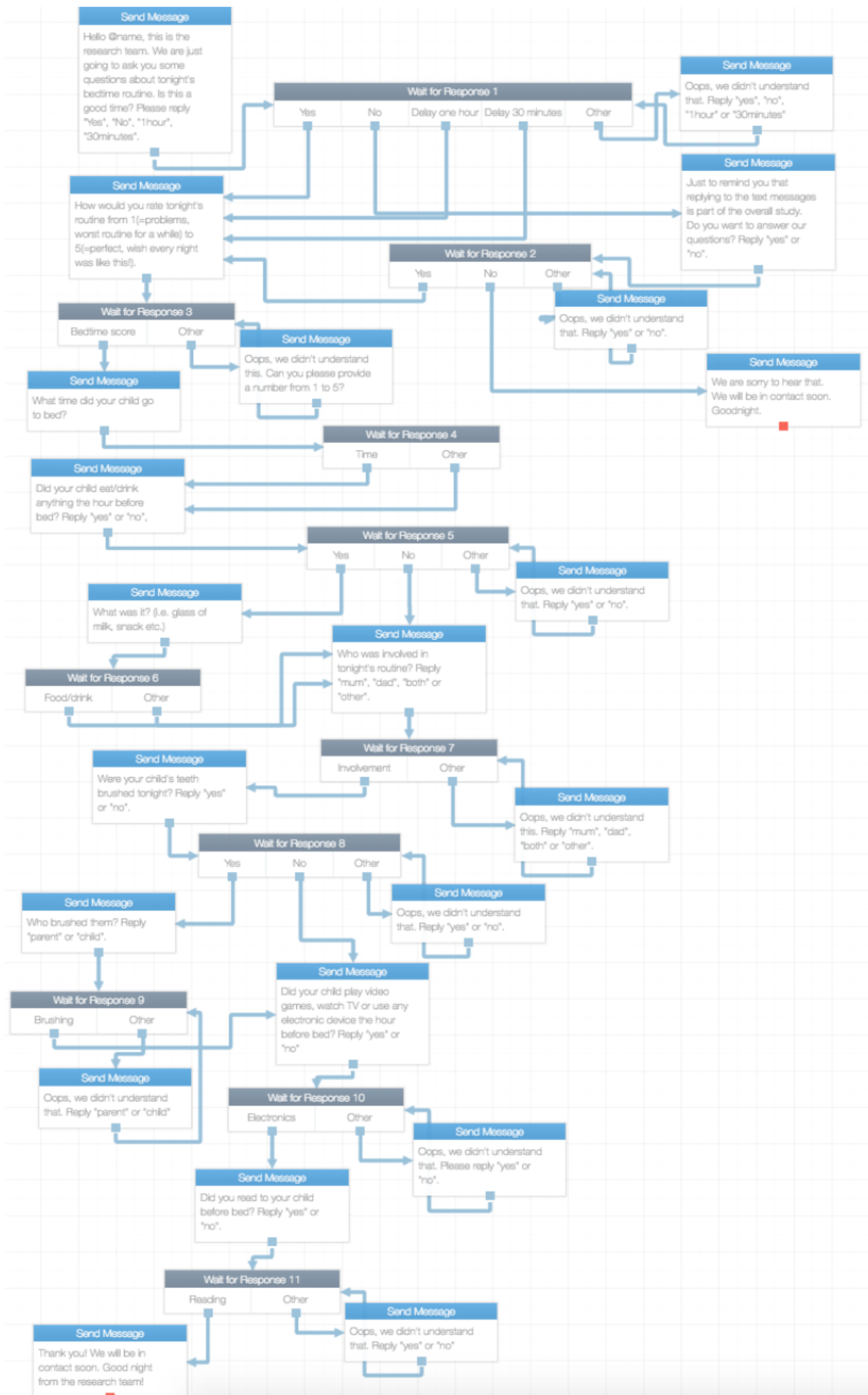

Supplement: Multimedia Appendix 2 [file publichealth_v6i4e15524_app2.pdf]
